# Evaluation of three sets of advanced backcrosses of eggplant with wild relatives from different gene pools under low N fertilization conditions

**DOI:** 10.1093/hr/uhad141

**Published:** 2023-07-18

**Authors:** Gloria Villanueva, Mariola Plazas, Pietro Gramazio, Reyes D Moya, Jaime Prohens, Santiago Vilanova

**Affiliations:** Instituto de Conservación y Mejora de la Agrodiversidad Valenciana, Universitat Politècnica de València, Camino de Vera 14, 46022, Valencia, Spain; Instituto de Conservación y Mejora de la Agrodiversidad Valenciana, Universitat Politècnica de València, Camino de Vera 14, 46022, Valencia, Spain; Instituto de Conservación y Mejora de la Agrodiversidad Valenciana, Universitat Politècnica de València, Camino de Vera 14, 46022, Valencia, Spain; Instituto de Conservación y Mejora de la Agrodiversidad Valenciana, Universitat Politècnica de València, Camino de Vera 14, 46022, Valencia, Spain; Instituto de Conservación y Mejora de la Agrodiversidad Valenciana, Universitat Politècnica de València, Camino de Vera 14, 46022, Valencia, Spain; Instituto de Conservación y Mejora de la Agrodiversidad Valenciana, Universitat Politècnica de València, Camino de Vera 14, 46022, Valencia, Spains

## Abstract

The development of new cultivars with improved nitrogen use efficiency (NUE) is key for implementing sustainable agriculture practices. Crop wild relatives (CWRs) provide valuable genetic resources for breeding programs aimed at achieving this goal. In this study, three eggplant (*Solanum melongena*) accessions together with their advanced backcrosses (ABs; BC3 to BC5 generations) were evaluated for 22 morpho-agronomic, physiological, and NUE traits under low nitrogen (LN) fertilization conditions. The ABs were developed with introgressions from the wild relatives *Solanum insanum*, *Solanum dasyphyllum*, and *Solanum elaeagnifolium*. The AB population comprised a total of 25, 59, and 59 genotypes, respectively, with overall donor wild relative genome coverage percentages of 58.8%, 46.3%, and 99.2%. The three *S. melongena* recurrent parents were also evaluated under control (normal) N fertilization. Reduction of N fertilization in the parents resulted in decreased chlorophyll content-related traits, aerial biomass, stem diameter, and yield and increased NUE, nitrogen uptake efficiency (NUpE), and nitrogen utilization efficiency (NUtE). However, the decrease in yield was moderate, ranging between 62.6% and 72.6%. A high phenotypic variation was observed within each of the three sets of ABs under LN conditions, with some individuals displaying improved transgressive characteristics over the recurrent parents. Using the single primer enrichment technology 5 k probes platform for high-throughput genotyping, we observed a variable but high degree of recurrent parent genome recovery in the ABs attributable to the lines recombination, allowing the successful identification of 16 quantitative trait loci (QTL). Different allelic effects were observed for the introgressed QTL alleles. Several candidate genes were identified in the QTL regions associated with plant growth, yield, fruit size, and NUE-related parameters. Our results show that eggplant materials with introgressions from CWRs can result in a dramatic impact in eggplant breeding for a more sustainable agriculture.

## Introduction

Enhancing crop productivity is a fundamental objective in agriculture, and remarkable advancements have been achieved in this area since the beginning of the 20th century. This has been accomplished, in part, through the widespread usage of nitrogen (N) as a fertilizer [1]. However, excessive use of N fertilization can lead to negative environmental impacts, such as groundwater and surface water contamination, loss of biodiversity, increased greenhouse gas emissions, and ozone layer depletion [2–4]. In addition, synthetic N fertilizers require large amounts of energy to be produced [5]. Therefore, to mitigate these negative consequences, selection and development of new varieties with improved crop nitrogen use efficiency (NUE) is a major objective of plant breeding for a more sustainable agriculture [6, 7].

The usage of plant genetic resources is essential for implementing breeding programs to address challenges associated with changes in climatic conditions. In this context, crop wild relatives (CWRs) are of great relevance because they possess inherent adaptations to a wide range of adverse natural conditions [8]. However, the direct usage of CWRs in breeding programs is often impractical due to the presence of unfavorable traits and genetic barriers. Therefore, the development of advanced backcrosses (ABs) is a viable breeding strategy that expands the available genetic diversity by incorporating genomic fragments of CWR genomes into a mostly cultivated genetic background [9]. Furthermore, ABs are useful for the detection of quantitative trait loci (QTL) by associating phenotypic variation with specific regions of the genome.

Eggplant (*Solanum melongena* L.), also known as aubergine or brinjal, is a widely cultivated vegetable crop, belonging to the subgenus *Leptostemonum* of the Solanaceae family [10]. It is one of the most important solanaceous crops, ranking second only to tomato (*Solanum lycopersicum* L.) [11]. The recent development of genomic tools specific for eggplant, such as high-throughput genotyping platforms [12] and high-quality eggplant genome assemblies [13–16], among others, has facilitated genomic studies on this crop.

Eggplant wild relatives (CWRs) are classified into the primary (GP1), secondary (GP2) and tertiary (GP3) gene pools, based on their level of crossability with the cultivated species. Interspecific hybrids, ABs, and introgression lines (ILs) have been obtained by using several of these CWRs [17–19].

Among the CWRs for which ABs have been developed, *Solanum insanum* L. belongs to GP1 and is considered the wild ancestor of the common eggplant (*S. melongena*). This species grows in a wide range of environmental conditions, including infertile soils, and is naturally distributed throughout South and Southeast Asia, Madagascar, and Mauritius [20]. Among the many eggplant secondary gene pool (GP2) species, *Solanum dasyphyllum* Schumach. & Thonn is part of the Anguivi clade of the *Leptostemonum* subgenus and is considered the wild progenitor of the gboma eggplant (*Solanum macrocarpon* L.), an African cultivated eggplant [21]. Some studies have shown that *Solanum insanum*, *Solanum dasyphyllum*, and their interspecific hybrids with eggplant exhibit enhanced drought [22, 23] and salinity tolerance [24–26]. Another CWR of interest is the American species *Solanum elaeagnifolium* Cav., which is native to Northern Mexico and the United States and that can thrive in a wide range of climatic conditions, including semiarid areas, being a globally invasive plant [27, 28]. The development of backcrosses of *S. elaeagnifolium* with eggplant has been reported, making available a previously unexploited gene pool for eggplant breeding [29]. In addition, *S. elaeagnifolium* is a potential source for developing new varieties with enhanced drought tolerance [30] and adaptation to low-N inputs [31].

In the present work, we evaluated morpho-agronomic and composition traits of three *S. melongena* accessions (MEL5, MEL1, and MEL3) under two N fertigation conditions and three sets of ABs of these three accessions with introgressions from eggplant wild relatives *S. insanum*, *S. dasyphyllum*, and *S. elaeagnifolium* under low-N conditions. The study results provide valuable information in the identification of potential materials for eggplant breeding under low-N fertilization. Furthermore, detection of QTLs was made possible through the association of phenotyping data and the availability of high-density genotyping data of the ABs individuals.

## Results

### Genomic characterization

**Figure 1:**
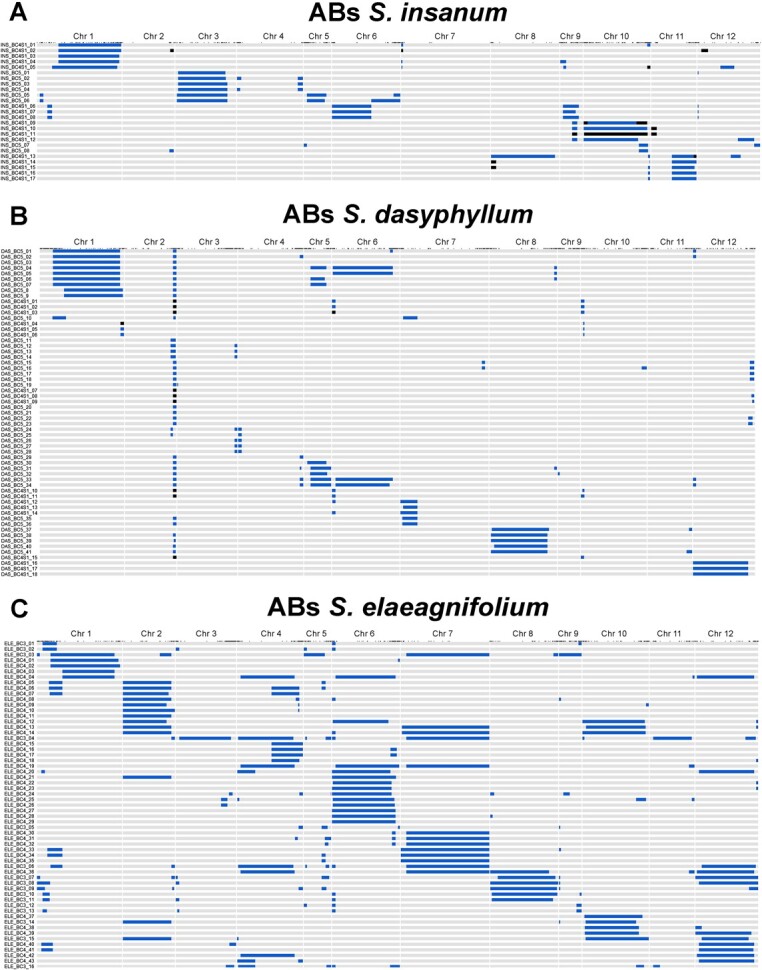
Graphical genotypes of ABs lines of *S. insanum* (A; n = 25), *S. dasyphyllum* (B; n = 59), and *S. elaeagnifolium* (C; n = 59) assessed for the present experiment. Each row corresponds to the ABs codes and genotypes and the columns indicate the chromosomes. Heterozygous introgressions are colored in blue, homozyogous introgressions are colored in black, and the genetic background of each recurrent parent (*S. melongena* MEL5, MEL1, and MEL3, respectively) are colored in grey.

Genome coverage of the donor wild relatives in the whole sets of ABs was 58.8% for *S. insanum*, 46.3% for *S. dasyphyllum*, and 99.2% *S. elaeagnifolium* when both heterozygous and homozygous introgressions are considered (Figure 1). Selection of ABs set of *S. insanum* prioritized a proper representation of chromosomes 1 (86.8%), 3 (80.9%), 6 (100%), 9 (78.4%), 10 (100%), and a region of chromosome 11 (34.8%) with several individuals per introgression for its evaluation (Figure 1A). The set of 59 selected ABs of *S. dasyphyllum* included most of chromosomes 1 (84.8%), 5 (86.3%), 6 (89.9%), 8 (89.1%), and 12 (98.9%), plus an introgression at the end of chromosome 2 (11.5%) and another one at the beginning of chromosome 7 (20.2%) (Figure 1B). For the 59 ABs of *S. elaeagnifolium*, a high percentage of total genome coverage (in heterozygosis) was present for chromosomes 1, 2, 4, 6, 7, 8, 10, and 12, with several individuals for each large introgression (Figure 1C).

The percentage of recovered genetic background from the recurrent parent is on average higher in the set of ABs of *S. insanum* (between 88.3% and 98.3%) and *S. dasyphyllum* (between 97.1% and 99.6%), whereas in the ABs of *S. elaeagnifolium* the percentage of recovery of the recurrent parent is lower (between 69.3% and 98.7%). These differences in the percentages of recovery can be attributed to the varying generations of backcrossing used in each set of ABs. The ABs of *S. insanum* and *S. dasyphyllum* involve individuals from the BC5 and BC4S1 generations, respectively, which are further along in the backcrossing process compared with the ABs of *S. elaeagnifolium*, which consist of individuals from the BC3 and BC4 generations.

### Characterization of recurrent parents and ABs

Overall, significant differences were detected between N treatments in *S. melongena* recurrent parents (MEL5, MEL1, and MEL3) for plant and composition traits, except for anthocyanin content in leaves (P-Anth) in MEL5 and carbon content in stem (C-Stem) in MEL1 (Tables 1, 2, and 3) *S. melongena* individuals cultivated under NN conditions had higher chlorophyll content in leaf (P-Chl), nitrogen balanced index (P-NBI), aerial biomass (P-Biomass), stem diameter (P-Diam), yield (Yield), total number of fruits per plant (F-Number), and N and C content in leaves, fruits, and stem (N-Leaf, C-Leaf, N-Fruit, C-Fruit, N-Stem, C-Stem) than plants under LN conditions. In addition, a significant decrease in values of flavonol and anthocyanin content in leaves (P-Flav, P-Anth), NUE, NUpE, and NUtE were observed in NN plants (Table 1). MEL5 showed the highest significant differences between treatments in yield (Yield, 3.7-fold) and total number of fruits (F-Number, 5.0-fold), with higher values under NN conditions, and the lowest differences in NUE (13.2-fold), NUpE (10.0-fold), and NUtE (1.3-fold), with highest values under LN conditions (Table 1). On the other hand, MEL1 presented the greatest differences between treatments for N content in the different plant parts, with higher values in NN than in LN for leaf (N-Leaf, 3.0-fold), fruit (N-Fruit, 2.0-fold), and stem (N-stem, 3.6-fold) (Table 3).

Some differences were observed between recurrent parents in N treatments for fruit shape and size traits (Table 2). Fruit pedicel length (F-PedLength) was statistically higher in MEL 5 cultivated under LN conditions than under NN conditions, and the same was observed for fruit length (F-Length) and fruit mean weight (F-Weight) in MEL1 and MEL5. For fruit calyx length (F-CaLength) and fruit width (F-Width) no statistically significant differences were detected (Table 2).

No significant differences were observed between each set of ABs and its corresponding recurrent parent cultivated under LN conditions for plant traits, except for P-Anth in the set of ABs of *S. elaeagnifolium*, being the values significantly higher in MEL3 (1.1-fold) (Table 1). The same results were observed for fruit traits, except for F-Weight in the set of ABs of *S. dasyphyllum*, which displayed significantly lower mean values than its recurrent parent MEL1 individuals in F-Weight (1.2-fold) (Table 2). For composition traits, no statistically significant differences were detected (Table 3).

The distribution ranges for traits evaluated in the three sets of ABs were wider than those observed in the recurrent parents cultivated under LN conditions, and transgressive individuals were found for all traits. The recurrent parents MEL5, MEL1, and MEL3 cultivated under NN conditions showed a wider distribution range for P-Biomass, P-Diam, and yield (Table 1). In addition, the same results were observed in MEL5 for F-Number and nitrogen content in fruit and stem (N-Fruit and N-Stem), in MEL1 for N-Stem, and in MEL3 for the F-Number (Table 2, Table 3).

### Principal components analysis

**Figure 2:**
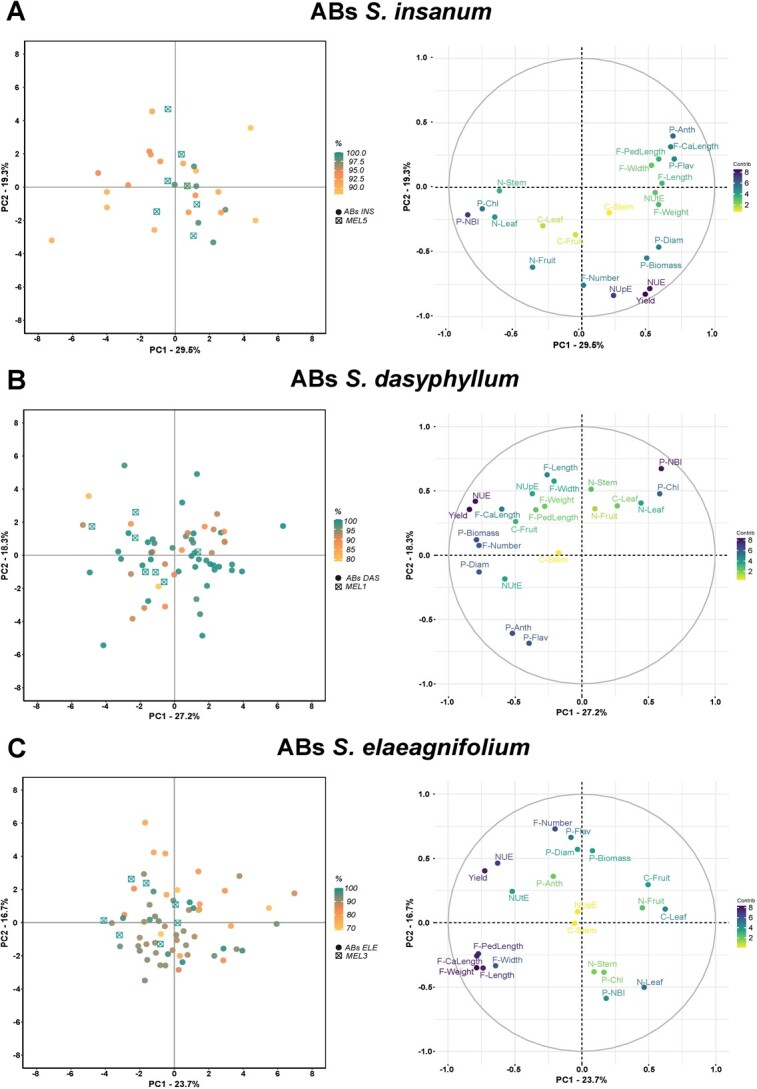
PCA score plot and loading plot based on the two first principal components of PCA performed for all traits of ABs of lines of *S. insanum* (A; n = 25), *S. dasyphyllum* (B; n = 59), and *S. elaeagnifolium* (C; n = 59) and recurrent parents MEL5 (n = 7), MEL1 (n = 7), and MEL3 (n = 7). The accessions are represented by different symbols according to the recurrent parent (MEL5, MEL1, and MEL3, respectively) and ABs of each line. Gradient of color in PCA score plot according to recovery percentage (%) from recurrent parent (100%: green to 80% (A); 70% (B); 90% (C): yellow). Gradient of color in PCA loading plot according to contribution proportion of each trait (8: dark blue to 2: yellow). The full name of each trait can be found in Table 5.

A PCA was performed with the traits evaluated for each of the sets of ABs with *S. insanum*, *S. dasyphyllum* and *S. elaeagnifolium* (Figure 2). Three groups of traits can be observed in common: one includes chlorophyll content-related traits (P-Chl, P-NBI) and nitrogen content in plant (N-Leaf and N-Stem); another group of correlated traits includes plant vigor traits (P-Biomass, P-Diam), yield, NUE, and the F-Number; and the third group involves fruit size traits (F-PedLength, F-CaLength, F-Length, F-Width, and F-Weight).

The PCA performed for the set of ABs of *S. insanum* and its recurrent parent *S. melongena* MEL5 revealed that the first two components accounted for 48.8% of the total variation observed, with PC1 and PC2 accounting for 29.5% and 19.3%, respectively (Figure 2A). The distribution of individuals in the PCA score plot showed that recurrent parent individuals of MEL5 were positioned along the central axis of PC1. Individuals with higher recovery percentages were located closer to the recurrent parentals. The first principal component displayed high negative correlation values with chlorophyll content-related traits (P-Chl, P-NBI) and positive correlations with P-Flav, P-Anth, and F-CaLength. NUpE, yield, NUE, and F-Number were highly negatively correlated with PC2, and P-Anth and F-CaLength were positively correlated to PC2 (Figure 2A, Table S3).

**Table 1:** Mean values and range of plant traits of *S. melongena* MEL5, MEL1, and MEL3 in LN and NN cultivation conditions and ABs of *S. insanum* (INS; *n* = 25), *S. dasyphyllum* (DAS; *n* = 59), and *S. elaeagnifolium* (ELE; n = 59) in LN cultivation conditions. The full name of each trait in the first column can be found in Table 5. For each trait, means with different letters are significant according to the Student–Newman–Keuls multiple range test (*P* < 0.05)

Plant traits	Mean/range	*S. melongena* MEL5 (*n* = 7)		ABs *S. insanum* INS (*n* = 25)	*S. melongena* MEL1 (*n* = 7)		ABs *S. dasyphyllum* DAS (*n* = 59)	*S. melongena* MEL3 (*n* = 7)		ABs *S. elaeagnifolium* ELE (*n* = 59)
		NN	LN	LN	NN	LN	LN	NN	LN	LN
P-Chl (μg cm^-2^)	Mean	45.8 b	37.7 a	36.1 a	38.2 b	29.9 a	30.2 a	37.4 b	25.4 a	27.7 a
	Range	41.0–49.7	35.8–41.7	30.2–42.4	35.7–41.7	27.5–33.7	24.3–37.1	33.4–43.6	24.4–27.4	20.7–33.6
P-Flav (μg cm^-2^)	Mean	1.5 a	1.9 b	1.9 b	1.7 a	2.5 b	2.4 b	1.7 a	2.5 b	2.5 b
	Range	1.3–1.6	1.8–2.2	1.5–2.4	1.5–1.8	2.3–2.6	1.9–2.8	1.4–1.8	2.4–2.6	2.2–2.8
P-Anth (μg cm^-2^)	Mean	0.20 a	0.23 ab	0.25 b	0.18 a	0.27 b	0.25 b	0.19 a	0.30 c	0.27 b
	Range	0.16–0.24	0.21–0.26	0.18–0.39	0.16–0.23	0.22–0.30	0.18–0.33	0.16–0.21	0.28–0.35	0.21–0.37
P-NBI	Mean	31.0 b	19.8 a	19.7 a	23.1 b	12.2 a	12.9 a	23.2 b	10.1 a	11.2 a
	Range	28.7–32.4	16.6–21.5	12.6–26.2	20.4–27.8	11.0–13.6	8.7–17.6	18.7–26.9	9.4–11.3	8.3–14.3
P-Biomass (kg FW)	Mean	1.9 b	0.4 a	0.4 a	2.2 b	0.4 a	0.4 a	1.4 b	0.3 a	0.3 a
	Range	1.2–2.4	0.2–0.6	0.2–0.6	1.5–3.0	0.2–0.6	0.1–0.7	0.7–2.5	0.2–0.4	0.1–1.0
P-Diam (mm)	Mean	20.4 b	11.2 a	11.9 a	22.6 b	13.3 a	13.1 a	19.2 b	12.7 a	13.1 a
	Range	15.9–29.7	9.5–12.2	9.0–14.5	16.2–33.0	11.3–15.0	7.9–20.4	14.6–24.3	10.7–14.5	9.5–18.5
Yield (g)	Mean	5881.9 b	1609.7 a	1609.6 a	6914.1 b	2584.6 a	2116.2 a	5464.4 b	1934.3 a	1564.6 a
	Range	4979.0–7464.0	994.0–2163.0	840.0–2264.0	5185.0–10601.0	1552.0–3553.0	793.0–4328.0	1612.0–8014.0	1257.0–2478.0	145.0–3395.0
NUE	Mean	7.9 a	104.1 b	103.7 b	7.5 a	144.2 b	114.8 b	7.6 a	122.3 b	97.9 b
	Range	5.8–10.6	57.8–142.1	52.3–167.1	5.9–11.4	88.7–216.2	38.8–297.9	2.5–11.1	74.9–157.1	12.9–203.4
NUpE	Mean	0.3 a	3.3 b	3.4 b	0.4 a	4.2 b	4.5 b	0.3 a	3.9 b	4.4 b
	Range	0.2–0.5	1.4–4.8	1.8–5.2	0.3–0.4	3.1–5.7	2.2–14.2	0.2–0.5	2.6–6.9	1.9–15.1
NUtE	Mean	24.2 a	32.5 b	30.3 b	21.5 a	34.5 b	27.1 ab	22.9 a	33.2 b	25.0 ab
	Range	20.2–30.4	25.6–42.6	20.7–43.0	16.9–26.9	28.9–42.0	7.0–46.4	15.3–28.5	22.8–45.2	2.9–56.3

**Table 2:** Mean values and range of fruit traits of *S. melongena* MEL5, MEL1, and MEL3 in LN and NN cultivation conditions and ABs of *S. insanum* (INS; *n* = 25), *S. dasyphyllum* (DAS; *n* = 59), and *S. elaeagnifolium* (ELE; *n* = 59) in LN cultivation conditions. The full name of each trait in the first column can be found in Table 5. For each trait, means with different letters are significant according to the Student–Newman–Keuls multiple range test (*P* < 0.05)

Fruit traits	Mean/range	*S. melongena* MEL5 (*n* = 7)		ABs *S. insanum* INS (*n* = 25)	*S. melongena* MEL1 (*n* = 7)		ABs *S. dasyphyllum* DAS (*n* = 59)	*S. melongena* MEL3 (*n* = 7)		ABs *S. elaeagnifolium* ELE (*n* = 59)
		NN	LN	LN	NN	LN	LN	NN	LN	LN
F-PedLength (mm)	Mean	35.7 a	43.3 b	39.3 ab	39	44.7	39.5	41.6	46.8	40.5
	Range	32.1–40.9	34.7–50.4	14.3–51.8	27.2–48.1	40.3–54.5	19.7–58.4	32.0–49.1	37.9–55.6	20.6–66.7
F-CaLength (mm)	Mean	25.9	27.3	26.9	35.7	40.1	35.4	45	41.9	37.5
	Range	21.8–37.5	24.3–29.1	11.7–33.5	31.9–39.7	35.6–43.0	16.1–44.0	38.0–47.7	30.7–51.6	17.3–51.9
F-Length (mm)	Mean	76.3 a	97.3 b	87.2 ab	71.8 a	90.6 b	84.7 b	100.1	93.8	85
	Range	64.0–86.5	71.4–118.2	33.5–106.0	57.3–87.5	69.0–114.5	30.3–114.3	84.6–122.2	70.6–115.5	37.0–132.4
F-Width (mm)	Mean	41.1	37.7	36	53.5	52.9	47.8	54.8	42.7	39.6
	Range	33.4–49.3	31.8–44.0	17.0–41.8	43.3–61.7	46.0–60.3	18.4–56.7	50.8–58.4	34.0–54.5	17.9–54.4
F-Number	Mean	198.7 b	39.7 a	42.7 a	115.7 b	35.7 a	36.5 a	87.7 b	32.9 a	32.6 a
	Range	126.0–268.0	27.0–58.0	22.0–66.0	93.0–143.0	25.0–49.0	17.0–78.0	32.0–118.0	19.0–49.0	12.0–82.0
F-Weight (g)	Mean	30.3 a	40.8 b	38.2 b	59.5 a	71.9 b	58.7 a	61.7	60.7	49.3
	Range	24.1–40.4	32.9–48.1	24.4–54.2	43.2–74.1	59.7–80.8	34.6–82.3	50.4–69.6	48.0–77.7	12.1–81.5

**Table 3:** Mean values and range of composition traits of *S. melongena* MEL5, MEL1, and MEL3 in LN and NN cultivation conditions and ABsof *S. insanum* (INS; *n* = 25), *S. dasyphyllum* (DAS; n = 59), and *S. elaeagnifolium* (ELE; n = 59) in low nitrogen (LN) cultivation conditions. The full name of each trait in the first column can be found in Table 5. For each trait, means with different letters are significant according to the Student–Newman–Keuls multiple range test (*P* < 0.05)

Composition traits	Mean/range	*S. melongena* MEL5 (*n* = 7)		ABs *S. insanum* INS (*n* = 25)	*S. melongena* MEL1 (*n* = 7)		ABs *S. dasyphyllum* DAS (*n* = 59)	*S. melongena* MEL3 (*n* = 7)		ABs *S. elaeagnifolium* ELE (*n* = 59)
		NN	LN	LN	NN	LN	LN	NN	LN	LN
N-Leaf (g/kg DM)	Mean	58.9 b	27.2 a	26.7 a	61.4 b	20.7 a	24.4 a	57.4 b	28.2 a	28.4 ± 5.0 a
	Range	51.1–64.0	22.7–33.8	16.3–34.5	52.8–67.4	17.5–23.8	14.2–33.2	51.6–63.4	25.1–33.6	17.6–40.4
C-Leaf (g/kg DM)	Mean	453.1 b	414.7 a	413.8 a	455.9 b	410.1 a	415.3 a	465.7 b	423.1 a	425.6 ± 18.3 a
	Range	436.0–464.0	400.0–433.0	388.0–435.0	450.0–463.0	398.0–429.0	394.0–437.0	458.0–480.0	407.0–436.0	385.0–471.0
N-Fruit (g/kg DM)	Mean	33.3 b	20.3 a	20.5 a	37.4 b	19.0 a	19.8 a	34.6 b	18.6 a	19.7 ± 3.3 a
	Range	28.4–40.1	17.4–22.6	15.9–24.0	31.6–44.8	14.6–22.1	13.8–27.2	31.9–37.6	14.4–27.2	13.6–31.2
C-Fruit (g/kg DM)	Mean	422.3 b	403.0 a	407.0 a	413.4 b	402.3 a	398.5 a	425.1 b	396.6 a	405.7 ± 13.4 a
	Range	410.0–436.0	384.0–428.0	368.0–419.0	409.0–425.0	382.0–420.0	357.0–424.0	402.0–446.0	348.0–415.0	376.0–441.0
N-Stem (g/kg DM)	Mean	31.8 b	10.3 a	10.8 a	37.1 b	10.2 a	10.3 a	29.9 b	10.2 a	11.2 ± 2.7 a
	Range	28.0–35.6	6.6–13.4	7.3–14.4	32.2–45.4	7.2–13.3	7.0–19.3	24.3–33.8	8.6–12.5	7.9–18.6
C-Stem (g/kg DM)	Mean	416.4 b	395.1 a	400.0 a	424.1	413	411.2	424.0 b	412.4 a	409.3 ± 9.2 a
	Range	414.0–420.0	389.0–410.0	387.0–415.0	417.0–431.0	403.0–419.0	388.0–494.0	415.0–432.0	398.0–423.0	387.0–430.0

For the set of ABs of *S. dasyphyllum* and its recurrent parent *S. melongena* MEL1, the first and the second principal components (PCs) accounted for 27.2% and 18.3%, respectively, of the variation (Figure 2B). The projection of individuals in the PCA score plot showed that the individuals of the recurrent parent MEL1 displayed a wide distribution, being intermingled with some ABs individuals. Individuals with different recovery percentages were distributed all over the graph. The first component was highly negatively correlated with yield, NUE, plant vigor traits (P-Biomass, P-Diam), and F-Number and positively with chlorophyll content-related traits (P-Chl, P-NBI). The second component was highly negatively correlated with flavonol and anthocyanin content in leaves (P-Flav and P-Anth) and positively with P-NBI and F-Length (Figure 2B, Table S3).

Regarding PCA performed for ABs of *S. elaeagnifolium* and its recurrent parent *S. melongena* MEL3, the first and the second components accounted, respectively, for 23.7% and 16.7%, of the observed variation (Figure 2C). The distribution of the individuals in the PCA score plot revealed a wide overall dispersion over the plot area, with most of the individuals with the lowest percentage of the recovered genetic background of the recurrent parent plotting apart from the recurrent parent MEL3 individuals. The composition traits carbon and nitrogen content in leaf (C-Leaf and N-Leaf) and C-Fruit were positively correlated with PC1, whereas some size-related fruit traits (F-Weight, F-CaLength, F-PedLength, and F-Length) were highly negatively correlated with PC1. On the other hand, the second component was highly positively correlated with P-Flav and F-Number and negatively correlated with P-NBI and N-Leaf (Figure 2C, Table S3).

### Correlations among traits

**Figure 3:**
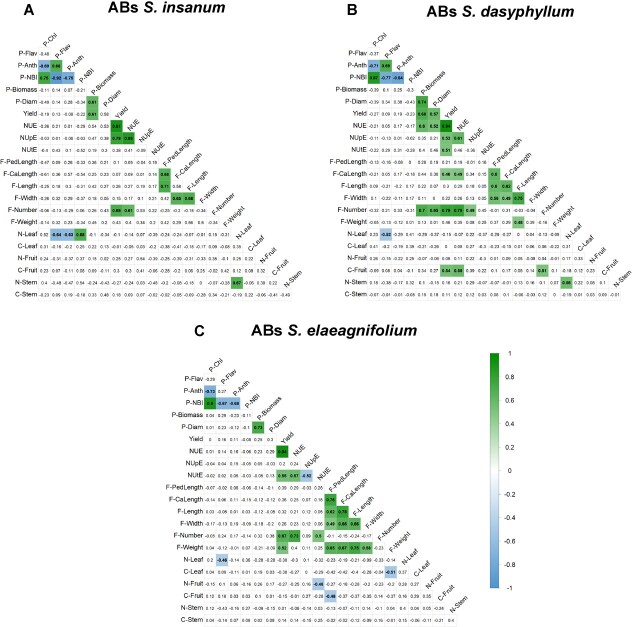
Pearson correlations among traits evaluated ABs of *S. insanum* (A; n = 25), *S. dasyphyllum* (B; n = 59) and *S. elaeagnifolium* (B; n = 59). Only significant correlations at *P* < 0.01 according to the Bonferroni tests are colored. Color scale from green (positive correlations) to blue (negative correlations). The full name of each trait can be found in Table 5.

Significant Pearson linear correlations among traits evaluated were found in the three sets of ABs of eggplant with *S. insanum*, *S. dasyphyllum*, and *S. elaeagnifolium* (Figure 3). For plant traits, negative correlations common to all ABs sets were observed among pigment content in leaves (P-Chl, P-Anth) and between P-NBI and anthocyanin and flavonol content in leaf (P-Anth, P-Flav). Positive correlations were detected between P-Chl and P-NBI. Traits related to plant vigor P-Biomass and P-Diam were positively correlated (r > 0.6). In addition, yield, NUE, and total number of fruits (F- Number) were positively correlated. Regarding fruit shape and size traits, shared positive correlations were found among F-PedLength and F-Width with F-CaLength and F-Length. For composition traits, N-Leaf showed a common significant negative correlation with P-Flav (Figure 3).

The set of ABs of *S. insanum* shared different significant correlations with the set of ABs of *S. dasyphyllum*. In this way, a positive correlation was observed between P-Flav and P-Anth (r > 0.6) in both sets (Figure 3A, B). Yield, in addition to showing significant positive correlations with NUE, was also correlated with P-Biomass and NUpE. N-Leaf and N-Stem also displayed a shared positive correlation in both ABs sets. On the other hand, several correlations among traits were shared between the ABs of *S. dasyphyllum* and the ABs of *S. elaeagnifolium*. In both sets, yield showed a positive correlation with NUtE (r > 0.5) (Figure 3B, C), whereas for fruit traits, F-length was positively correlated with F-CaLength and F-Weight, and F-PedLength was positively correlated with F-Width.

In addition, each set of ABs showed specific correlations. In this way, for ABs of *S. insanum*, N-Leaf displayed a positive correlation with P-Anth and a negative with P-NBI (Figure 3A). Regarding specific significant correlations found in ABs of *S. dasyphyllum*, NUE and F-Number were positively correlated with plant vigor-related traits (P-Biomass and P-Diam) (r > 0.7) (Figure 3B). NUE was also correlated with F-CaLength and C-Fruit, and F-Number showed a positive correlation with NUpE. In addition, yield showed positive correlations among F-CaLength and C-Fruit in this set of ABs. Finally, for ABs set of *S. elaeagnifolium*, NUtE showed positive correlations with other nitrogen use efficiency parameters (NUE and NUpE) and with F-Number, and a negative correlation with N-Fruit (Figure 3C). F-Weight was positively correlated with yield and traits related to fruit size (F-PedLength, F-CaLength, F-Length, and F-Width) and negatively correlated with C-Leaf. In addition, in this set of ABs F-PedLength was negatively correlated with C-Fruit.

### Detection and effect of putative quantitative trait loci

**Figure 4:**
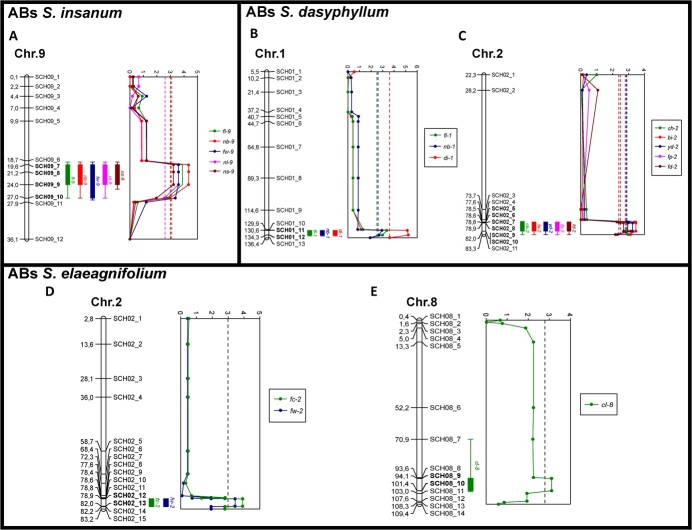
Physical map and LOD score of chromosomes with putative QTLs. A, ABs of *S. insanum*; B-C, ABs of *S. dasyphyllum*; and D–E, ABs of *S. elaeagnifolium*. Dotted lines indicate the LOD score thresholds of each QTL, with corresponding chromosome positions indicated on the physical map.

**Figure 5:**
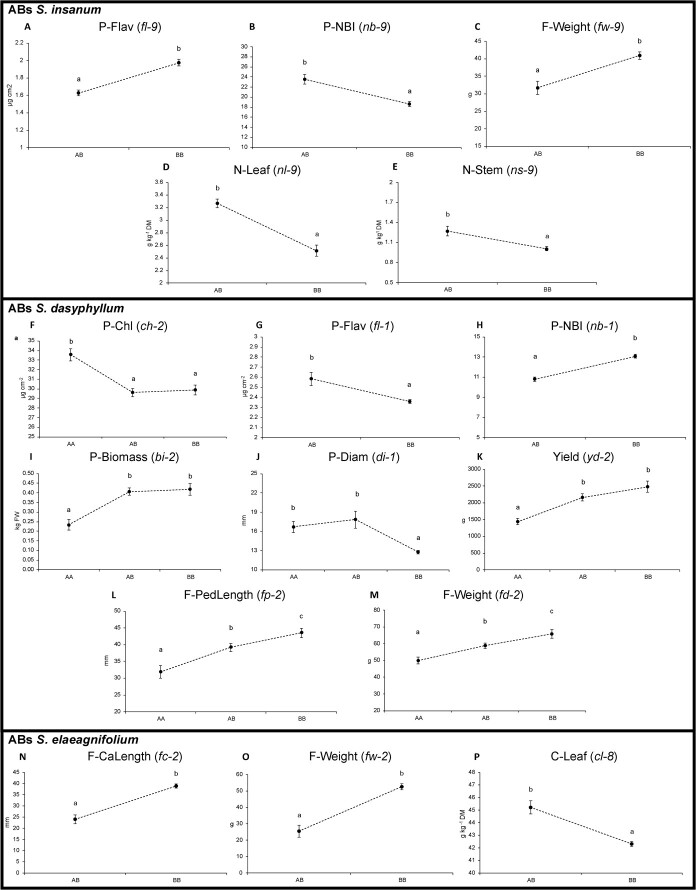
Effect plots for putative QTLs based on allelic distributions determined at the peak marker of the QTL. Three possible genotypes are indicated on the *x*-axis (A = wild parent, B = recurrent parent, AB = hybrid). A–E, ABs of *S. insanum*; F–M, ABs of *S. dasyphyllum*; and N–P, ABs of *S. elaeagnifolium*. The *y*-axis represents mean values of each trait for each genotype. Error bars represent standard error of the mean (SEM). For each trait, means with different letters are significant according to the Student–Newman–Keuls multiple range test (*P* < 0.05).

A total of 16 putative significant QTLs were found in the analysis of the three different sets of ABs (Table 4). Five QTLs (flavonol leaf content, *fl-9*; nitrogen balanced index, *nb-9*; fruit mean weight, *fw-9*; nitrogen content in leaf, *nl-9*; and nitrogen content in stem; *ns-9*) were found in ABs of *S. insanum* at the same position on chromosome 9 (Table 4, Figure 4A). QTLs for flavonol leaf content (*fl-9*) and for mean fruit weight (*fw-9*) presented similar effects (Figure 5A, C). In contrast, opposing effects were observed for QTLs associated with nitrogen balance index (*nb-9*), nitrogen leaf content (*nl-9*), and nitrogen stem content (*ns-9*), which presented significant higher values in individuals with heterozygous introgression (Figure 5B, D, E). Regarding ABs of *S. dasyphyllum*, eight of them were identified. Three QTLs were detected at the same position on chromosome 1 (Table 4, Figure 4B). QTL for flavonol leaf content (*fl-1*) presented significant higher values in individuals with heterozygous introgression, whereas an opposite QTL effect was observed for nitrogen balanced index (*nb-1*) (Figure 5G, H). For stem diameter (*di-1*), a dominance of the *S. melongena* allele decreasing the values was observed (Figure 5J). On chromosome 2, five QTLs were located at the same position (Table 5, Figure 4C). Chlorophyll leaf content (*ch-2*) presented dominance of the *S. dasyphyllum* allele, which displayed significant higher values, whereas an opposite QTL effect was observed for biomass (*bi-2*) and yield (*yd-2*) (Figure 5F, I, K). For fruit pedicel length (*fp*-2) and fruit mean weight (*fd-2*) incomplete dominance was observed with a negative allelic effect of *S. dasyphyllum* introgression on the values (Figure 5L, M). The analysis of ABs of *S. elaeagnifolium* allowed the detection of two QTLs for fruit traits, namely fruit calyx length (*fc-2*) and fruit mean weight (*fw-2*) located at the same position on chromosome 2 (Table 5, Figure 4D), showing very similar effects with significant lower values in individuals with heterozygous introgression (Figure 5N, O). A QTL was also detected on chromosome 8 (Figure 4E), displaying significant higher carbon content in leaf (*cl-8*) corresponding to the *S. elaeagnifolium* heterozygous introgression (Figure 5P).

**Table 4:** List of putative QTLs detected for traits in ABs of *S. melongena* (MEL5, MEL1, and MEL3) with *S. insanum*, *S. dasyphyllum*, and *S. elaeagnifolium*. QTL name, chromosome, position (Mb) their genomic location, and LOD score

ABs	Trait	QTL	Chr.	Position (Mb.)	LOD Score
ABs *S. insanum*	*Plant traits*				
	Flavonol leaf content (P-Flav)	*fl-9*	9	19.6–27.0	3.89
	Nitrogen balanced index (P-NBI)	*nb-9*	9	19.6–27.0	4.35
	*Fruit traits*				
	Fruit mean weight (F-Weight)	*fw-9*	9	19.6–27.0	3.6
	*Composition traits*				
	Nitrogen content in leaf (N-Leaf)	*nl-9*	9	19.6–27.0	3.6
	Nitrogen content in stem (N-Stem)	*ns-9*	9	19.6–27.0	3.23
ABs *S. dasyphyllum*	*Plant traits*				
	Chlorophyll leaf content (P-Chl)	*ch-2*	2	78.5–83.3	3.38
	Flavonol leaf content (P-Flav)	*fl-1*	1	130.6–134.3	3.37
	Nitrogen balanced index (P-NBI)	*nb-1*	1	130.6–134.3	2.94
	Aerial biomass (P-Biomass)	*bi-2*	2	78.5–83.3	3.47
	Stem diameter (P-Diam)	*di-1*	1	130.6–134.3	5.23
	Yield	*yd-2*	2	78.5–83.3	3.26
	*Fruit traits*				
	Fruit pedicel length (F-PedLength)	*fp-2*	2	78.5–83.3	3.48
	Fruit mean weight (F-Weight)	*fd-2*	2	78.5–83.3	3.15
ABs *S. elaeagnifolium*	*Fruit traits*				
	Fruit calyx length (F-CaLength)	*fc-2*	2	78.4–83.2	3.92
	Fruit mean weight (F-Weight)	*fw-2*	2	78.4–83.2	3.44
	Composition traits				
	Carbon content in leaf (C-Leaf)	*cl-8*	8	94.0–101.4	3.11

### Identification of candidate genes

The search for candidate genes in the ‘67/3’ eggplant reference genome assembly (V3 version) [14] allowed identification of several potential candidate genes that may be associated to putative QTLs detected in this study. For the QTLs detected in ABs of *S. insanum* related to nitrogen content in plants, a gene encoding NITRATE TRANSPORTER 1/PEPTIDE TRANSPORTER (NRT1/PRT) FAMILY (NPF) proteins (SMEL_009g328470) was identified and mapped to the corresponding region of the detected QTL on chromosome 9. Regarding the QTLs detected on chromosome 2 associated with plant growth, yield, and fruit size parameters in ABs of *S. dasyphyllum* and *S. elaeagnifolium*, two distinct potential candidate genes were found to be possibly associated to these traits. One gene (SMEL_002g164700) encodes a PIN-FORMED (PIN) 8 auxin efflux transporter, whereas another gene (SMEL_002g167520) encodes a Myb-related protein 306 (MYB306), which is a transcription factor (TF) involved in anthocyanin regulation [32]. In addition, within the same region on chromosome 2, a gene (SMEL_002g164340) was detected that encodes an NPF protein, which could also be associated with the same traits.

## Discussion

The use of populations with CWR introgressions, such as ABs, enables the usage of variation present in the CWRs, facilitating the development of crop varieties that are suitable for sustainable agriculture practices [9]. ABs populations increase the precision in detection of QTLs because the different genotypes share a common genetic background that differs only in one or a few introgressed genomic fragments of the donor species [31]. Effective fertilizer management is crucial for improving sustainability, with NUE being a critical breeding goal due to its significant impact on economic and environmental factors [7, 33, 34]. Several studies have been conducted to improve NUE through breeding in different crops, particularly in cereals and potato [35]. However, until recently, few efforts have been made to address this issue in eggplant in recent years [31, 36–39].

In this study, three sets of ABs of eggplant wild relatives from different gene pools, namely *S. insanum* (GP1), *S. dasyphyllum* (GP2), and *S. elaeagnifolium* (GP3) were evaluated under the same conditions for the first time. The availability of eggplant ABs with introgressions in different chromosomes provided an overview of the potential of wild species for breeding when evaluated under LN input abiotic stress conditions. In addition, recurrent parental lines of *S. melongena* (MEL5, MEL1, and MEL3) were tested under LN and NN input, providing insights into the effect of N in the different traits in cultivated eggplant.

Previous studies evaluated NUE by conducting hydroponic culture in different Solanaceae species, including tomato [40, 41], potato [42, 43], and eggplant [36, 39]. Other works investigated NUE using soil cultivation methods [31, 44–46] or combining different abiotic stresses [47–49]. Moreover, different approaches have been documented for the evaluation of NUE [50]. The use of pots and the automatic N fertilization system used here allowed for more controlled conditions to evaluate the impact on different traits under LN input. This approach allows greater control of the experimental conditions because all plants are subjected to the same fertilization and substrate than soil cultivation.

The study revealed significant differences in plant and composition traits of *S. melongena* recurrent parents cultivated under different N treatments. Generally, plants grown under NN conditions showed higher values in chlorophyll content and lower values in flavonol and anthocyanin content. These traits, which have been reported to correlate with nitrogen content in plant leaves, can be effectively measured using proximal optical sensors for nitrogen, thereby enabling optimized management of vegetable crop cultivation [51, 52]. The results also indicated that plants grown under NN conditions displayed higher values for the studied traits such as aerial biomass, stem diameter, yield, total number of fruits per plant, and nitrogen and carbon content in plant and fruits. These findings are consistent with previous reports that demonstrated the impact of different nitrogen fertilization treatments on eggplant [37, 38, 53]. In contrast to Mauceri *et al*. [37], the LN treatment resulted in much higher NUE, NupE, and NUtE values than under NN. Similar results were found by Rosa-Martínez *et al*. [38] suggesting that established fertilization practices may not always be the most efficient or sustainable approach to eggplant cultivation. Instead, carefully managed fertigation with reduced nitrogen inputs can improve NUE, resulting in higher yield per unit of N fertilization applied. Moreover, no significant differences were observed for most traits between each set of ABs and their respective recurrent parents under LN conditions. However, the wider distribution ranges for some traits observed within sets of ABs, which is in agreement with Villanueva *et al*. [31], suggests that these materials may be of interest for enhancing the overall performance and variability of eggplant. The set of ABs used in this study do not cover the entire genome of wild eggplant relatives, indicating that further investigation into unexplored regions could result in significant findings for eggplant breeding under LN conditions.

The results of the PCA analysis revealed a relatively wide distribution of the ABs in the PCA plot. Despite the general trend of ABs genotypes, with lower recovery percentages being distributed separately from the recurrent parents, it is also observed that some accessions with high recovery percentages are distributed separately, whereas others with low recovery percentages are closely located to the recurrent parents. This suggests that, in general, a high recovery of the recurrent genome background in ABs is needed to have a general phenotype similar to the recurrent parent, although there are exceptions that may have great interest for breeding.

Breeding programs can benefit from correlations observed among similar traits in each set of ABs because these can help in predicting the phenotype of specific traits, requiring the assessment of fewer traits. Correlations established between traits evaluated by leaf clip meter Dualex® are consistent with results in previous studies [54, 55]. Similarly, associations identified between traits linked to plant vigor, such as plant biomass and stem diameter, and those related to yield, NUE, and number of fruits per plant, as well as those associated with fruit morphology, are in agreement with several studies [31, 38, 56, 57]. Notably, some relevant correlations related to nitrogen content in plant were observed, including negative correlations between nitrogen content and flavonol and anthocyanin content in leaves, as well as positive intercorrelation between nitrogen content in leaves and stem. In addition, differences in correlations among NUE, NUpE, NUtE, and other traits, such as fruit calyx length or those related to plant vigor, were found between sets of ABs.

The identification of 16 putative significant QTLs across three different sets of ABs demonstrates the potential of genetic variation present in wild eggplant relatives. QTLs located on chromosome 2 for *S. dasyphyllum* and *S. elaeagnifolium*, associated with plant growth, yield, and fruit size parameters, may suggest the presence of genetic linkage or a pleiotropic locus, which is consistent with results reported in previous studies [15, 38, 58, 59]. Furthermore, QTLs detected on chromosomes 2 and 9 for fruit pedicel and calyx length, as well as fruit weight, have also been identified in different collections and eggplant populations in earlier studies [15, 38, 58, 60–62]. For traits measured with the DUALEX® optical leaf clip meter, including chlorophyll, flavonol, and NBI, novel QTLs in eggplant were identified on chromosomes 1 and 2 for *S. dasyphyllum* and chromosome 9 for *S. insanum*. In addition, for composition traits, a novel QTL for carbon content in leaf was found on chromosome 8 in ABs of *S. elaeagnifolium*, whereas two novel QTLs for nitrogen content in leaf and stem were located on chromosome 9 in ABs of *S. insanum*. In comparison, Rosa-Martínez [38] reported QTLs for carbon leaf content on chromosomes 1, 5, and 10, and for leaf nitrogen content on chromosomes 4 and 9 in *S. melongena* ILs with eggplant wild relative *S. incanum* as the donor parent. Interestingly, both studies detected QTLs associated with leaf nitrogen content on chromosome 9, suggesting possible common underlying genetic factors.

In the identified QTL regions associated with plant growth, yield, fruit size, and nitrogen-related parameters in ABs of *S. insanum*, *S. dasyphyllum*, and *S. elaeagnifolium*, several potential candidate genes have been detected. On chromosomes 2 and 9, genes encoding NPF proteins (SMEL_002g164340; SMEL_009g328470) were identified. These NPF proteins are involved in nitrate uptake and transport of various substrates in plants, contributing to diverse biological processes [63, 64]. Consequently, they may potentially influence nitrogen content and other nitrogen-related parameters in plants. Furthermore, on chromosome 2, a candidate gene (SMEL_002g164700) was identified, which encodes a PIN-FORMED (PIN) 8 auxin efflux transporter. This transporter is crucial for auxin distribution and affects a wide range of developmental processes in plants [65]. In the same chromosomal region, another candidate gene (SMEL_002g167520) was identified, encoding a Myb-related protein 306 (MYB306), This protein is a TF involved in anthocyanin regulation [32] and has been suggested to potentially influence both anthocyanin accumulation and fruit size in eggplant [66].

## Conclusions

This study highlights the potential of wild eggplant relatives for breeding under LN conditions by evaluating three sets of ABs and their recurrent parental lines. The findings reveal significant differences in plant, fruit, and composition traits in response to different nitrogen levels. Furthermore, we observed notable phenotypic variation among the ABs lines under LN fertilization, revealing the potential of introgression materials for genetic improvements in eggplant. The availability of genotyped lines with genetic variation allowed for the identification of putative QTLs. These insights may contribute to the development of breeding strategies aimed at improving eggplant productivity, quality, and nitrogen use efficiency under LN conditions, supporting sustainable agriculture practices.

## Materials and methods

### Plant material

Three *S. melongena* accessions (MEL5, MEL1, and MEL3) and three sets of ABs of these accessions with, respectively, the eggplant wild relatives *S. dasyphyllum* DAS1, *S. elaeagnifolium* ELE2, and *S. insanum* INS1 were used for the current study [19]. Each of the three sets of ABs has gone through several rounds of recombination in the successive backcrossings performed toward the three recurrent parents. Selection of the recombinant ABs genotypes was performed based on single primer enrichment technology (SPET) molecular markers to maintain an overall coverage of the wild genome donor while increasing the genetic background of the recurrent domesticated parent. For the set of *S. insanum* ABs (INS1 ×MEL5), 25 ABs genotypes were used, of which eight were from the fifth backcross generation (BC5) and 17 of the first selfing of the fourth backcross generation (BC4S1). In the case of the *S. dasyphyllum* ABs set (MEL1 ×DAS1), a total of 59 genotypes were used, 41 of them being from the BC5 generation and 18 of the BC4S1 generation. Finally, for the *S. elaeagnifolium* ABs set (MEL3 ×ELE2), 59 ABs genotypes were used, of which 16 were from the third backcross generation (BC3) and 43 were from the fourth backcross generation (BC4).

### DNA extraction and genotyping

Extraction of genomic DNA of the three recurrent parents and ABs individuals was performed following the SILEX DNA extraction method [67]. Isolated DNA was evaluated for quality and integrity by 0.8% agarose gel electrophoresis and spectrophotometric ratios 260:280 and 260:230 and quantified by a Qubit® 2.0 Fluorometer (Thermo Fisher Scientific, Waltham, MA, USA). Diluted DNA samples were genotyped using the eggplant 5 k SPET platform consisting of 5093 probes [12]. Single nucleotide polymorphisms (SNPs) were filtered with Tassel software (version 5.2 Standalone [68];) by using a minimum count value of 97%, a minimum allele frequency (MAF) higher than 5%, a maximum heterozygosity proportion of 70%, and a minimum distance between adjacent sites of 2000 pb. After filtering, the number of discriminant SNPs between parents was 826, 1195, 2114, and for *S. insanum*, *S. dasyphyllum*, and *S. elaeagnifolium* ABs, respectively.

**Table 5:** Plant, fruit, and composition traits evaluated in the *S. melongena* MEL1, MEL3, and MEL5 recurrent parents, and their respective ABs genotypes with *S. dasyphyllum*, *S. elaeagnifolium*, and *S. insanum*, together with abbreviations and units used in the present study

**Trait**	**Abbreviation**	**Units**
*Plant traits*		
Chlorophyll leaf content	P-Chl	μg cm^−2^
Flavonol leaf content	P-Flav	μg cm^−2^
Anthocyanin leaf content	P-Anth	μg cm^−2^
Nitrogen balanced index	P-NBI	—
Aerial biomass	P-Biomass	kg FW^a^
Stem diameter	P-Diam	mm
Yield	Yield	g plant ^−1^
Nitrogen use efficiency	NUE	—
Nitrogen uptake efficiency	NUpE	—
Nitrogen utilization efficiency	NUtE	—
*Fruit traits*		
Fruit pedicel length	F-PedLength	mm
Fruit calyx length	F-CaLength	mm
Fruit length	F-Length	mm
Fruit width	F-Width	mm
Total number of fruits per plant	F-Number	—
Fruit mean weight	F-Weight	g
*Composition traits*		
Nitrogen content in leaf	N-Leaf	g kg^−1^ DM^b^
Carbon content in leaf	C-Leaf	g kg^−1^ DM
Nitrogen content in fruit	N-Fruit	g kg^−1^ DM
Carbon content in fruit	C-Fruit	g kg^−1^ DM
Nitrogen content in stem	N-Stem	g kg^−1^ DM
Carbon content in stem	C-Stem	g kg^−1^ DM

a FW: fresh weight. ^b^ DM:dry matter

### Cultivation conditions

Plants were grown during the summer season (July to October 2020) in an open field plot located on the campus of the Universitat Politècnica de València (GPS coordinates: latitude, 39° 28′ 55” N; longitude, 0° 20′ 11” W; 7 m a.s.l.). ABs individuals and recurrent parentals lines were randomly distributed in 17-l pots with coconut fiber, spaced 150 cm between rows and 70 cm within rows. Irrigation and fertilization were applied with a drip irrigation system.

The recurrent parents *S. melongena* MEL5, MEL1, and MEL3 were cultivated under two different nitrogen fertilization conditions, namely low (LN) and normal nitrogen (NN) treatments. Seven plants of each *S. melongena* accession together with ABs individuals of each set were cultivated under LN conditions, whereas seven plants of each *S. melongena* were cultivated under NN conditions.

A physicochemical and composition analysis of coconut fiber was performed before the transplant. Parameters were evaluated following the procedures described in van Reeuwijk [69] and are shown in Table S1. A chemical composition analysis of water was performed before adding fertilizers. The intake water was slightly basic with low content of nitrates, nitrites, phosphates, ammonium, magnesium and potassium, moderate content of sulphates and calcium, and high content of sodium (Table S2).

Fertilization solutions were prepared based on the substrate composition and the intake water analyses. The LN solution was prepared by adding 1.5 mM H_3_PO_4_ (Antonio Tarazona SL., Valencia, Spain), 4.85 mM K_2_SO_4_ (Antonio Tarazona SL.), 0.58 mM MgSO_4_ (Antonio Tarazona SL.) plus 0.025 l/m^3^ of a microelements Welgro Hydroponic fertilizer (Química Massó S.A., Barcelona, Spain) containing boron (BO33-; 0.65% p/v), copper (Cu-EDTA; 0.17% p/v), iron (Fe-DTPA; 3.00% p/v), manganese (Mn-EDTA, 1.87% p/v), molybdenum (MoO42-; 0.15% p/v), and zinc (Zn-EDTA; 1.25% p/v). NN solution included the components listed previously with the addition of 7.2 mM NH4NO3 to the intake water. The pH of the solutions was adjusted to 5.5–5.8 with 23% HCl (Julio Ortega SL., Valencia, Spain).

### Phenotypic trait evaluation

Plants were evaluated for a total of 22 plant, fruit, and composition traits (Table 5). A DUALEX® optical leaf clip meter (Force-A, Orsay, France) was used for measuring the chlorophyll, flavonol, anthocyanin contents, and nitrogen balance index (NBI®) in leaves [70, 71]. Data were obtained as the mean of 10 measurements in the upper and lower side of five leaves of each plant. At the end of the trial, stem diameter was measured with a caliper at the base of the stem and aerial biomass was immediately weighed after cutting the base of the stem with a Sauter FK-250 dynamometer (Sauter, Balingen, Germany). Subsequently, they were dried at room temperature; the leaves were separated from the stems, ground, and weighed after drying in an oven at 70°C to constant dry weight. The total number of fruits of each plant was harvested for determining yield. Nitrogen uptake efficiency (NUpE) was calculated as the total content of N in fruit, stem, and leaves divided by N supplied with the irrigation solution per plant; nitrogen utilization efficiency (NUtE) was calculated as total fruit yield in dry weight (yield [DM]) divided by the total content of N in fruit, stem, and leaves. NUE was the result of the multiplication of NUpE and NUtE [6, 72, 73].

For fruit traits, pedicel length, calyx length, fruit length, and width were determined as the mean of at least three fruits per plant harvested at the commercially mature stage (i.e. physiologically immature). Fruits traits evaluated were measured with a caliper, and their abbreviations and units are included in Table 5.

To determine fruit N and carbon (C) content, at least five commercially mature fruits per plant were harvested, peeled, chopped, and frozen in liquid N_2_ and stored at −80°C. Subsequently, the frozen samples were lyophilized, ground until turned into fine powder, and homogenized. Dry powder of leaves, stem, and fruits was measured in samples of 0.5 g of freeze-dried powder. The analysis of N content was performed using the Dumas method with a TruSpec CN elemental analyzer (Leco, MI, USA). C content was calculated from the measurements of carbon dioxide (CO_2_) using an infrared detector [74]. Certified reference standards of different N and C concentrations were used for the quantification.

### Data analysis

For plant, fruit, and composition data of each ABs set and the recurrent parents (*S. melongena* MEL5, MEL1, and MEL3), mean, standard deviation (SD), range values, and coefficient of variation (CV, %) were calculated. Analysis of variance (ANOVA) was performed to detect significant mean differences between the two N cultivation conditions in the recurrent parents and between each set of ABs and its corresponding recurrent parent in the LN conditions. Significant differences were detected with the Student–Newman–Keuls multiple range test at *P* < 0.05 using Statgraphics Centurion 18 software (StatPoint Technologies, Warrenton, VA, USA).

For each set of ABs and its recurrent parent cultivated in the same conditions (LN), a principal component analysis (PCA) was performed. Pairwise Euclidean distances were calculated for the analysis of each PCA using R package stats [75] of the R statistical software [76]. The PCA score and loading plots were drawn using R packages ggplot2 [77] and RColorConesa [78]. In addition, Pearson pairwise correlation coefficients were calculated among traits for each set of ABs and *S. melongena* parents cultivated under LN conditions. Their statistical significance was evaluated using a Bonferroni correction at *P* < 0.01 [79] using R packages psych [80] and corrplot [81].

### Quantitative trait loci detection and candidate gene identification

Detection of QTLs was performed for each set of ABs using the single QTL model for genome-wide scanning of the R package *R/qtl* [82] of R statistical software v4.1.0 [76]. The threshold of logarithm of odds (LOD) score was established at the 0.05 probability level for significant QTLs. For each putative QTL detected, allelic effects were calculated by establishing significant differences between the means of each genotype with the Student–Newman–Keuls multiple range test (*P* < 0.05).

To identify potential candidate genes within each QTL region, a search was conducted using the ‘67/3’ eggplant reference genome assembly (V3 version) [14]. This search was performed through the Sol Genomics Network database (http://www.solgenomics.net).

## Supplementary Material

Web_Material_uhad141

## Data Availability

Relevant data can be found within the paper and its supporting materials. All data of this study are available from the corresponding author upon reasonable request.
